# Disseminated fusariosis and endogenous fungal endophthalmitis in acute lymphoblastic leukemia following platelet transfusion possibly due to transfusion-related immunomodulation

**DOI:** 10.1186/1471-2415-11-30

**Published:** 2011-11-02

**Authors:** Tan Aik Kah, Ku Chui Yong, Ropilah Abdul Rahman

**Affiliations:** 1 Department of Ophthalmology, Faculty of Medicine and Health Sciences, Universiti Malaysia Sarawak (UNIMAS) Lot 77, Seksyen 22, Kuching Town Land District, Jalan Tun Ahmad Zaidi Adruce, 93150 Kuching, Sarawak, Malaysia; 2 Department of Ophthalmology, Faculty of Medicine, Universiti Kebangsaan Malaysia Medical Centre (UKMMC) Jalan Yaacob Latif, Bandar Tun Razak, Cheras, 56000 Kuala Lumpur, Malaysia

## Abstract

**Background:**

To report a case of disseminated fusariosis with endogenous endophthalmitis in a patient with acute lymphoblastic leukemia. Transfusion-associated immune modulation secondary to platelet transfusion could play an important role in the pathophysiology of this case.

**Case Presentation:**

A 9 year-old male with acute lymphoblastic leukemia complicated by pancytopenia and disseminated Intravascular coagulation was given platelet transfusion. He developed disseminated fusariosis and was referred to the ophthalmology team for right endogenous endophthalmitis. The infection was controlled with aggressive systemic and intravitreal antifungals.

**Conclusion:**

Patients with acute lymphoblastic leukemia are predisposed to endogenous fungal endophthalmitis. Transfusion-associated immune modulation may further increase host susceptibility to such opportunistic infections.

## Background

Endogenous fungal endophthalmitis is a serious sight threatening condition, occur mostly in immunocompromised patients. Patients with acute lymphoblastic leukemia require aggressive polychemotherapy with high risk of bone marrow suppression. Frequent transfusion of blood product may result in transfusion-associated immune modulation (TRIM) which further increases host susceptibility to opportunistic infection. TRIMs are mostly associated with the transfusion of allogenic white blood cells. We report a case of disseminated fusariosis and endogenous fungal endophthalmitis in a patient with acute lymphoblastic leukemia (ALL) after platelet transfusion.

## Case Presentation

A 9 year old male was diagnosed of precursor B-CALLA positive ALL in December 2006, and was on maintenance therapy since February 2007. He had testicular relapse in June 2009 and central nervous system (CNS) involvement in March 2010. He was treated according to ALL R3 protocol phase III intensification which consist intravenous vincristine 1.5 mg, intravenous cytarabine 3,000 mg/m^2 ^and intrathecal methotrexate 12 mg.

In June 2010, he developed pancytopenia with low grade fever (37-38 degree Celsius). Haemoglobin was 8.7 g/dL, red cell count 3.18 × 10^12^/L, white cell count 0.2 × 10^9^/L and platelet count 19 × 10^9^/L.

The Disseminated Intra-Vascular Coagulation (DIVC) screening test was positive; Prothrombin time (PT) 16.9 seconds, International Normalized Ratio (INR) 1.42, Activated Partial Thromboplastin Time (APTT) 67.8 seconds, APTT ratio 1.75, fibrinogen level 8.79 g/dL and D-Dimer 2.67 ug/ml. The patient was given urgent transfusion of irradiated apheresis platelet, fresh frozen plasma and cryoprecipitate.

**Figure 1:**
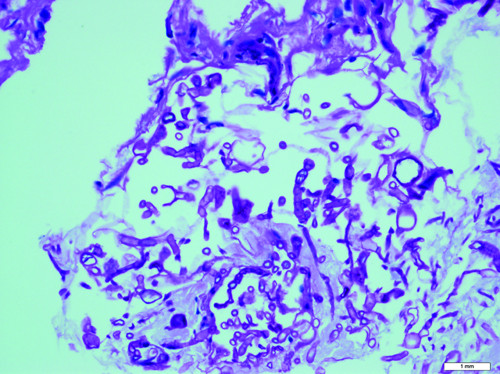
**Histopathological specimen of the skin (PAS stain)**.

**Figure 2:**
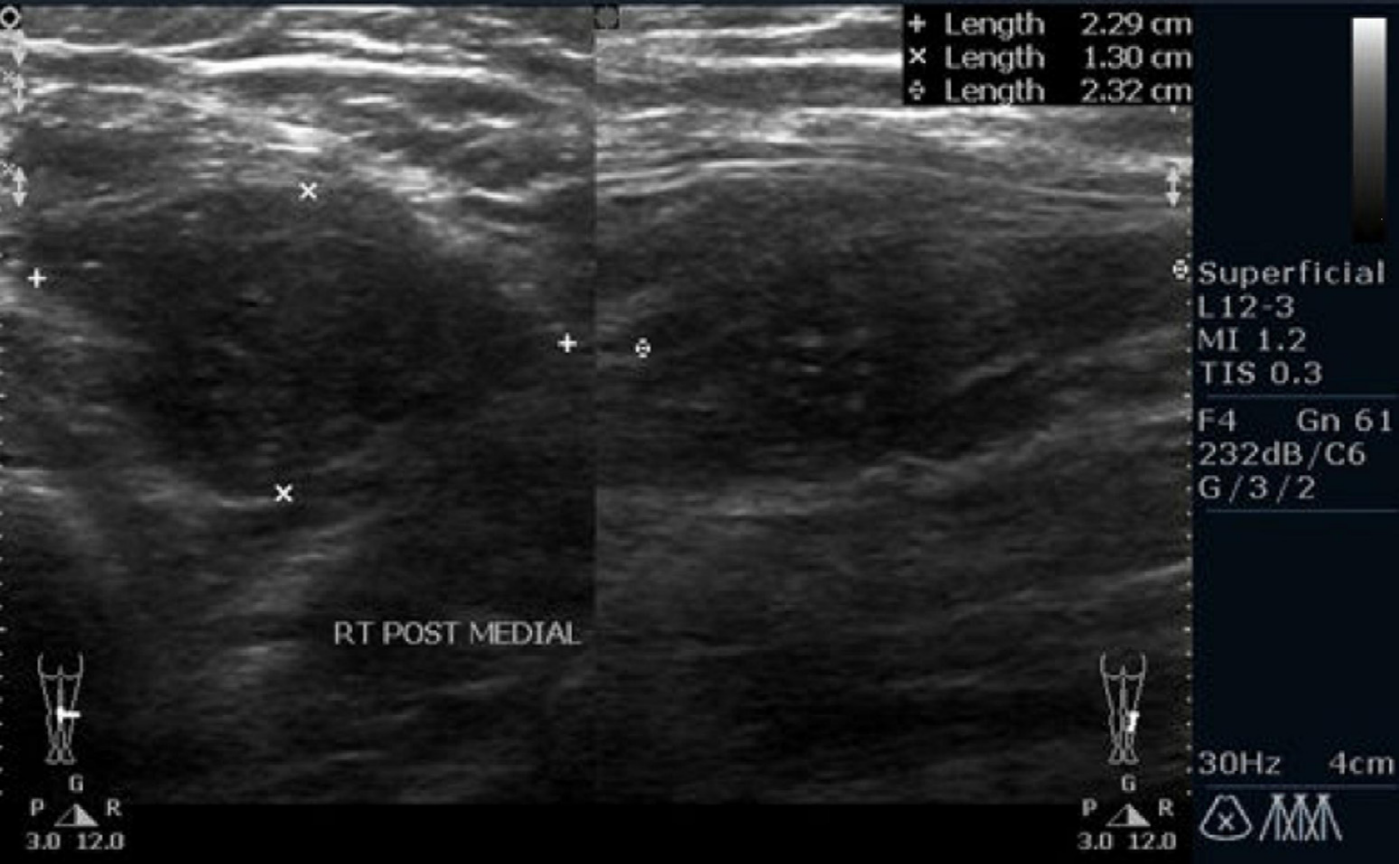
**Ultrasonography of right leg**. Hypoechoic lesion (abscess) at subcutaneous tissue of posteromedial aspect of the right leg.

**Figure 3:**
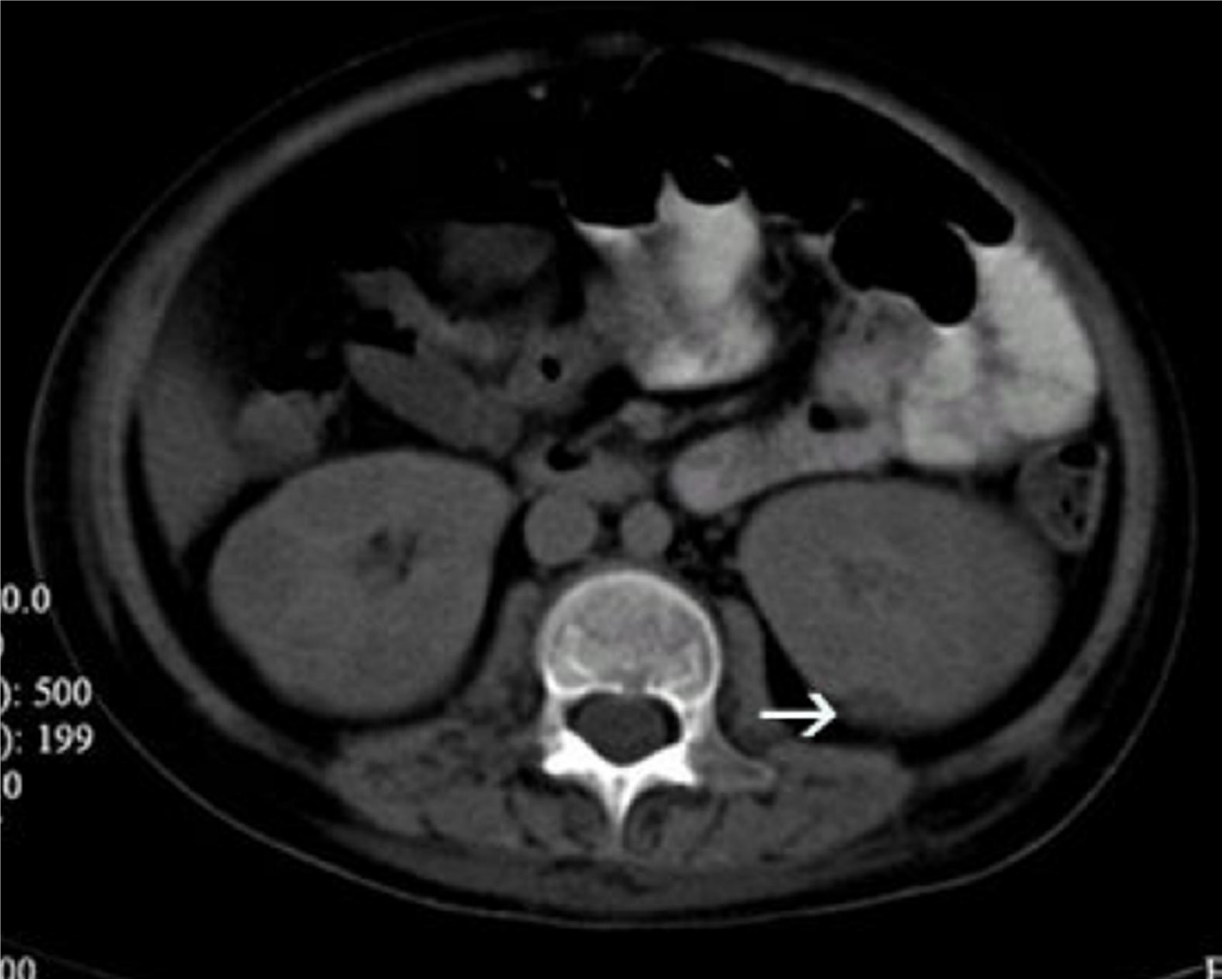
**CT abdomen (transverse view)**. Small hypodense lesion(abscess) in the posterior cortex of the lower pole of the left kidney (white arrow).

**Figure 4:**
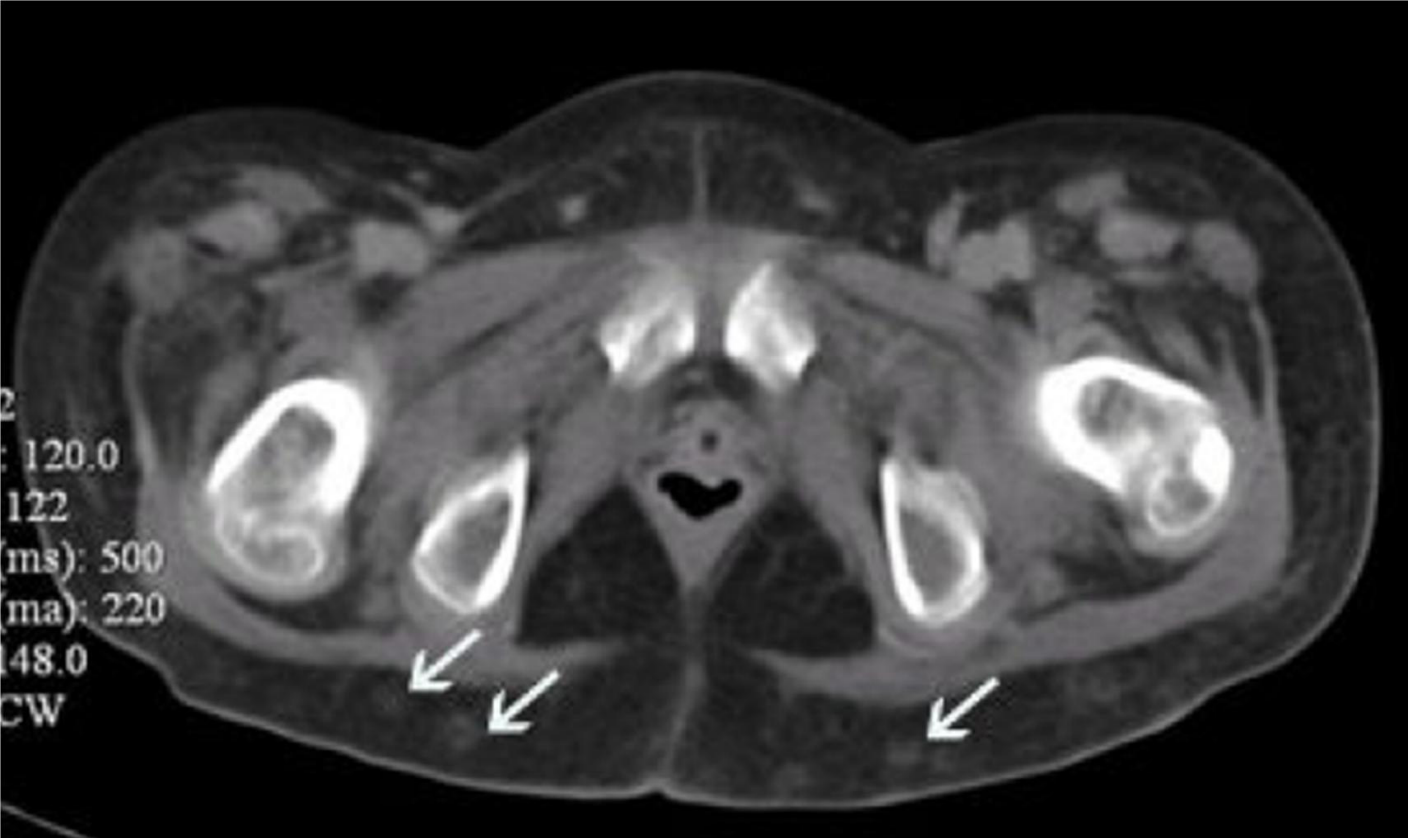
**CT abdomen (transverse view)**. Multiple small subcutaneous abscesses(white arrows).

Within 48 hour of platelet transfusion, the patient developed generalized tender, papular rash. The rash was initially thought to be of viral origin. Skin biopsy revealed dermis infiltration by septated, non-pigmented fungal hyphae (Figure 1). Fungal antigen tests for *aspergillus *and *candida *antigen were negative. Ultrasonography of both legs showed disseminated fungal granuloma (Figure 2). Computer Tomography (CT) abdomen showed an abscess at the posterior cortex of the lower pole of the left kidney (Figure 3). There were also multiple subcutaneous abscesses (Figure 4). The patient was treated with intravenous Amphotericin B Cholesterol Dispersion (ABCD) 5 mg/kg 8 hourly. Blood culture was positive for *Fusarium *species. Sensitivity to antifungals was not performed due to technical difficulties. Subcutaneous granulocyte-colony stimulating factor (G-CSF) 125 mcg daily was given to counteract persistent neutropenia. After five days, the white cell count increased from 0.4 × 10^9^/L to 7.1 × 10^9^/L, while the platelet count was 110 × 10^9^/L.

Three weeks after the onset of disseminated fungal infection, the patient was still treated with intravenous ABCD. He was referred to the ophthalmic team for redness of left eye of two days duration. Visual acuity was unable to be documented reliably. There was no relative afferent pupillary defect (RAPD). Two septic iris nodules appeared at the pupillary margin; intraocular pressure (IOP) was 11 mm Hg. Red reflex was reduced due to severe vitritis and numerous vitreous fungal balls. Optic disc was hyperaemic with tortuosity of the retinal vessels. The right eye was not involved. The diagnosis was left endogenous fungal endophthalmitis, presumed to be due to *Fusarium *species. He was treated with immediate intravitreal voriconazole 100 mcg/0.1 ml, intravitreal vancomycin 1 mg/0.1 ml and intravitreal ceftazidime 2.5 mg/0.1 ml. Vitreous and aqueous sample contained pus cells without any identifiable microorganisms and culture was negative. Response to treatment was slow. Two weeks after the first injection, a second intravitreal voriconazole 100 mcg/0.1 ml was given.

**Figure 5:**
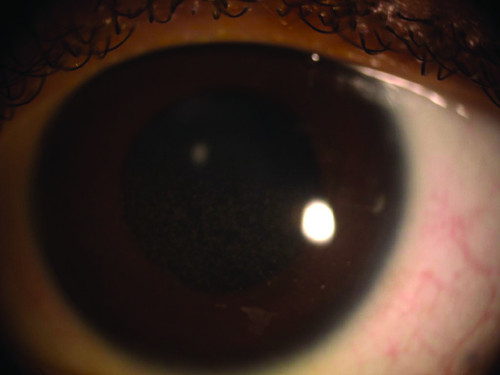
**Anterior segment of the left eye**.

**Figure 6:**
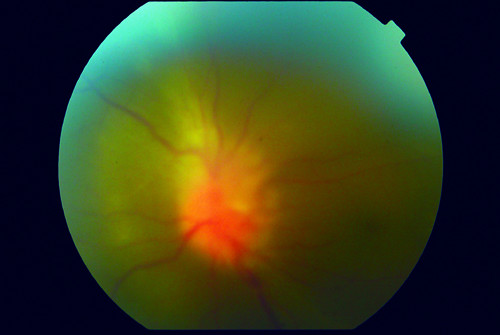
**Fundus photography of the left eye**.

Two months after the second intravitreal voriconazole, the visual acuity was 6/24 (decimal notation: 0.25) for the left eye and 6/6 (decimal notation: 1.0) for the right eye. There were numerous keratic precipitates and iris pigments on the corneal endothelium (Figure 5). No cells and flare in the anterior chamber. Vitreous opacity and optic disc hyperaemia reduced, but with the development of epiretinal membrane and subretinal fibrosis (Figure 6). The white cell counts remain low at the vicinity of 2 - 4 × 10^9^/L after resolution of the systemic fungal infection.

## Discussion

Children with acute lymphoblastic leukemia (ALL) who relapse after successful frontline induction and consolidation therapy can achieve long-term event-free survival with aggressive polychemotherapy. A second remission is achieved by more intensive short-course elements containing high-dose methotrexate and high-dose cytarabine [1]. For treatment or prevention of CNS leukemia, intrathecal therapy is given during intensive treatment, and CNS irradiation is administered after the end of intensive chemotherapy [2]. In testicular relapse, local irradiation or orchidectomy is performed [3]. Bone marrow suppression and pancytopenia is a known complication of both ALL itself and cancer chemotherapy. Neutropenia increases patients' susceptibility to opportunistic infections. To overcome neutropenia, subcutaneous injection of Granulocyte colony-stimulating factor (G-CSF) is used to enhance the production of neutrophils within the bone marrow [4].

Blood transfusion is frequently needed for anemia and thrombocytopenia. Urgent platelet transfusion is required in this patient because of severe thrombocytopenia and DIVC.

Irradiated apheresis platelets were used. Irradiation removes leukocytes from the platelet. The major advantage of apheresis platelets is that enough platelets can be collected from a single donor to constitute a transfusion dose. The reduction in donor exposures by using apheresis platelets and the removal of leukocytes have the advantages of reducing transfusion-transmitted infections and the incidence of platelet alloimmunization.

The bacterial risk associated with platelet transfusions is high because platelets are stored at 22°C rather than at 4°C as are red cells [5]. However, the risk of contamination of any blood product with fungal element is very low. The development of fungal septicaemia within 48 hours of platelet transfusion in this patient could simply due to superinfection in a case of immunosuppression from ALL and immunosuppressive therapy. Alternatively platelet transfusion may lead to TRIM which increases the risk of endogenous fungal endophthalmitis above and beyond that posed by leukemia.

TRIM occur as a result of allogeneic blood transfusions (ABT) which induce clinically significant immunosuppression in recipients. Blood component transfusion causes recipient immunomodulation, with stimulation of certain immune responses and suppression of others, resulting in an impaired immune competence. TRIM has been linked to an improved clinical outcome in the setting of renal allograft transplantation. Possible deleterious TRIM-associated effects include an increased rate of cancer recurrence and of post-operative bacterial infection. TRIM is attributed to immunomodulatory and pro-inflammatory mechanisms. TRIM effects may be mediated by allogeneic mononuclear cells, white-blood-cell (WBC)-derived soluble mediators and soluble HLA peptides circulating in allogeneic plasma [6].

The presence of platelet-derived bioactive substances such as plasminogen activator inhibitor-1, tissue inhibitor of metalloproteinases-1, vascular endothelial growth factor and tissue factor, in platelet concentrates, may play an important role in TRIM [7]. It is now known that platelets possess cell membrane, cytoplasmic, and secrete CD40 ligand (CD40L). Previously thought to be involved only in hemostasis, platelets also function in innate and adaptive immunity and possess proinflammatory, as well as pro-thrombotic properties. They interact with other platelets, endothelial cells, lymphocytes, dendritic cells, and fibroblasts. Soluble forms of CD40L (sCD40L) in the human circulation are almost entirely derived from platelets. Elevated levels of CD40L are associated with vascular disease, abnormal clotting (thrombosis), lung injury, and autoimmune disease [8].

All antifungals are fungistatic, therefore neutrophilic recovery is important for the eradication of any disseminated fusariosis [9]. Early and aggressive local and systemic antifungal therapy is crucial, although the visual prognosis is generally poor. Binder et al identified visual acuity at diagnosis and the presence of hypopyon as factors associated with outcome [10]. In their series, all patients with visual acuity worse than 20/200 at diagnosis had final visual acuity of worse than 20/200. Two-thirds of patients with hypopyon at diagnosis had final visual outcomes worse than 20/200, whereas less than one-third of patients without hypopyon at diagnosis had final visual acuity worse than 20/200. There was no significant difference in outcomes for patients undergoing vitrectomy compared with those treated with intravenous antibiotics or antifungal agents. Vitrectomy was not performed for our patient due to unfavourable risk-to-benefit ratio. The risk of amblyopia is minimal and the patient was responding well to the combination of intravitreal voriconazole and systemic amphotericin B.

## Conclusion

This case report highlights the life-threatening and sight-threatening complications of acute lymphoblastic leukemia. TRIMs are mostly due to transfusion of allogenic white blood cells. However, other biological mediators of TRIM may exist. TRIMs are usually reported in debilitated patients. The evidence of TRIM in this case was masked by the presence of multiple confounding factors (absence of evidence); but the absence of evidence is not evidence of absence. As new evidence of platelet's capability to function in innate and adaptive immunity is emerging, platelet transfusion may increases the risk of endogenous fungal endophthalmitis above and beyond that posed by leukemia. Although the link between endogenous endophthalmitis and TRIM cannot be established by a single case report, it is our hope that this could path the way for future research in this area.

## Consent

Written informed consent was obtained from the patient's guardian for publication of this case report and any accompanying images. A copy of the written consent is available for review by the Editor-in-Chief of this journal.

## Competing interests

The authors declare that they have no competing interests.

## Authors' contributions

TAK, KCY, RAR treated the patient and in doing so acquired the case data; all were also involved with drafting of the manuscript.

All authors read and approved the final manuscript.

## Pre-publication history

The pre-publication history for this paper can be accessed here:

http://www.biomedcentral.com/1471-2415/11/30/prepub
